# A journey through the tinnitus universe

**DOI:** 10.1016/j.bjorl.2022.09.001

**Published:** 2022-09-14

**Authors:** Raquel Mezzalira

**Affiliations:** Universidade Estadual de Campinas (UNICAMP), Departamento de Otorrinolaringologia e Cirurgia de Cabeça e Pescoço, São Paulo, SP, Brazil

Tinnitus can be defined as the perception of sound in the absence of any external sound stimulus. According to the World Health Organization, approximately 15% of the world’s population have tinnitus and this prevalence increases with age.^1^ A population study carried out in the city of São Paulo report that 22% of the population had tinnitus. This prevalence triples in people over 65 years of age.^2^ This same study observed that tinnitus annoyance occurred in 64% of cases, and that it interfered with daily activities in 18% of individuals who reported the symptom.^2^ Hearing loss is associated with tinnitus in approximately 85–96% of cases.^3^ These numbers show that tinnitus does not affect the daily activities of most patients. However, it can have negative implications for the quality of life in part of the patients, with proven relationships with difficulty in attention and concentration, depression and even suicidal ideation. This group demands more care from health professionals, although all tinnitus should be inquired, regardless of its repercussions.

The study of tinnitus forces the physician to dive into the anatomical and physiological universe of the auditory pathways and their connections in an attempt to understand its origin and the reactions it can cause. The knowledge of the various neural networks involved in the different aspects of the symptom made tinnitus no longer seen only as an auditory phenomenon and started to be considered a sensory, cognitive and emotional disorder. The generation occurs mainly in the peripheral auditory pathways, detection occurs in the subcortical centers, but the perception takes place in the auditory cortex, which makes each patient experience different reactions to the symptom.^3^ Several comorbidities may be associated with tinnitus. Among the main ones, the otological, cardiovascular, metabolic, hormonal, neurological, somatosensory causes and diseases with somatopsychic characteristics stand out. This way, tinnitus may be the initial manifestation of several ear or systemic diseases. But depending on the impact of tinnitus on the affected individual, areas of the limbic system and autonomic nervous system that increase patient discomfort, may be activated. The cognitive behavioral process contributes to its severity through negative thoughts, selective attention, and hypervigilance. Therefore, it is essential to distinguish between tinnitus and the reactions caused by it. For that purpose, questionnaires assessing and quantifying tinnitus and its effects on the patient’s life can be used. Hearing and electrophysiological tests are necessary to assess the auditory pathways. Laboratory and imaging tests may also be helpful depending on the type of tinnitus.

The diagnosis of the factors involved in the origin of tinnitus is the greatest challenge in the management of these patients. Once this difficulty has been overcome, the next stage consists of an art: treatment. Science has been very generous on this point as several tinnitus treatment options are emerging. The art consists of knowing how to choose and use each one of them. Treatment must take into account not only the etiology but also the patient’s reactions and expectations regarding tinnitus.^4^ However, regardless of the therapy chosen, counseling is the main component of all of them. Explaining to the patient the reason for the tinnitus often reduce the negative reactions related to it. Subsequently, the treatment of the causes involved in the origin of tinnitus is necessary in an attempt to improve or even eliminate the symptom. But, unfortunately, this is not always possible because the cause cannot be treated or because the patient has a marked perception of tinnitus at the cortical level that makes it difficult to improve, but this does not mean that this perception cannot be addressed. In this case, therapeutic actions focus on creating methods to ignore information related to tinnitus. Cognitive behavioral therapy aims to identify and change the emotional meaning of tinnitus. Another possible approach is mindfulness, which has been demonstrating benefits on tinnitus, reducing annoyance and facilitating its acceptance by the patient. A third way to improve this situation is acupuncture, whose electrical discharge caused by needle stimulation triggers action potentials that influence the activity of the olivocochlear nucleus or modulate the connections between the auditory pathway and the limbic system and amygdala, which are areas related to the discomfort caused by tinnitus. Following this same way, there are still two other treatment modalities that act on the central auditory pathways, which are transcranial magnetic stimulation, which modulates neuroplasticity in cortical and thalamic areas, and neuromodulation, whose mechanism of action in tinnitus is attributed to interference on the inputs coming from of the central auditory pathways and their associated circuits that reach the cerebral cortex.

Due to the large association between tinnitus and hearing loss, improving acoustic stimulation to overcome the lack of stimulation of the auditory pathways is critical. In this sense, the use of hearing aids and/or sound generator are the first treatment option. In addition, environmental sounds also can help to minimize the effects of maladaptive cortical reorganization and reduce the perception of tinnitus. Sound therapy is also indicated for patients with tinnitus and normal hearing.

To date, there is no medication with specific indication for the treatment of tinnitus. However, many of them can be useful in controlling associated symptoms such as depression and anxiety or improving the function of the inner ear and their choice must take into account the patient’s needs. Some options are drugs that improve vascular supply, inner ear metabolism and neuronal function (ginkgo biloba, vitamin D, vitamin B12, zinc), drugs that act on ion channels (Gabapentin and Carbamazepine), drugs that act on neurotransmitters, (clonazepam, selective serotonin reuptake inhibitors, cyclobenzaprine) etc.^4^

The anatomical and functional associations between the ear, head, temporomandibular joint and neck are well known and are the origin of a very common subgroup of tinnitus: the somatosensory. The mechanism is complex and involves the disinhibition of dorsal cochlear nucleus activity through the serotonergic somatosensory pathway. This condition has been increasing nowadays with the home office and consequent inadequate postures. In addition, the incidence of bruxism has been increasing due to the stress caused by the Covid-19 pandemic and the acceleration of daily activities. In this context, the physical therapy approach to myofascial trigger points is the indicated treatment for this type of tinnitus.^5^

The variety of factors that can generate tinnitus and influence the degree of discomfort point to the need for an individualized and generally interdisciplinary approach. Maybe the best approach is to counseling and offer sounds. But there are several instruments available for treat tinnitus and improve the negative reactions caused by it. Unfortunately, some are only available in research centers, but the majority are accessible to all patients. It is up to the conductor to choose which instrument or instruments to use and to conduct the orchestra.
